# RAVAL trial: Protocol of an international, multi-centered, blinded, randomized controlled trial comparing robotic-assisted versus video-assisted lobectomy for early-stage lung cancer

**DOI:** 10.1371/journal.pone.0261767

**Published:** 2022-02-02

**Authors:** Yogita S. Patel, Waël C. Hanna, Christine Fahim, Yaron Shargall, Thomas K. Waddell, Kazuhiro Yasufuku, Tiago N. Machuca, Mauricio Pipkin, Jean-Marc Baste, Feng Xie, Andrea Shiwcharan, Gary Foster, Lehana Thabane

**Affiliations:** 1 Division of Thoracic Surgery, Department of Surgery, McMaster University, Hamilton, Ontario, Canada; 2 Division of Thoracic Surgery, Department of Surgery, University of Toronto, Toronto, Ontario, Canada; 3 Division of Thoracic and Cardiovascular Surgery, Department of Surgery, University of Florida, Gainesville, Florida, United States of America; 4 Division of Thoracic Surgery, Department of Surgery, Rouen Normandy University, Rouen Cedex, France; 5 Department of Epidemiology and Biostatistics, McMaster University, Hamilton, Ontario, Canada; 6 Funding Reform and Case Costing, St. Joseph’s Healthcare Hamilton, Hamilton, Ontario, Canada; Stanford University School of Medicine, UNITED STATES

## Abstract

**Background:**

Retrospective data demonstrates that robotic-assisted thoracoscopic surgery provides many benefits, such as decreased postoperative pain, lower mortality, shorter length of stay, shorter chest tube duration, and reductions in the incidence of common postoperative pulmonary complications, when compared to video-assisted thoracoscopic surgery. Despite the potential benefits of robotic surgery, there are two major barriers against its widespread adoption in thoracic surgery: lack of high-quality prospective data, and the perceived higher cost of it. Therefore, in the face of these barriers, a prospective randomized controlled trial comparing robotic- to video-assisted thoracoscopic surgery is needed. The RAVAL trial is a two-phase, international, multi-centered, blinded, parallel, randomized controlled trial that is comparing robotic- to video-assisted lobectomy for early-stage non-small cell lung cancer that has been enrolling patients since 2016.

**Methods:**

The RAVAL trial will be conducted in two phases: Phase A will enroll 186 early-stage non-small cell lung cancer patients who are candidates for minimally invasive pulmonary lobectomy; while Phase B will continue to recruit until 592 patients are enrolled. After consent, participants will be randomized in a 1:1 ratio to either robotic- or video-assisted lobectomy, and blinded to the type of surgery they are allocated to. Health-related quality of life questionnaires will be administered at baseline, postoperative day 1, weeks 3, 7, 12, months 6, 12, 18, 24, and years 3, 4, 5. The primary objective of the RAVAL trial is to determine the difference in patient-reported health-related quality of life outcomes between the robotic- and video-assisted lobectomy groups at 12 weeks. Secondary objectives include determining the differences in cost-effectiveness, and in the 5-year survival data between the two arms. The results of the primary objective will be reported once Phase A has completed accrual and the 12-month follow-ups are completed. The results of the secondary objectives will be reported once Phase B has completed accrual and the 5-year follow-ups are completed.

**Discussion:**

If successfully completed, the RAVAL Trial will have studied patient-reported outcomes, cost-effectiveness, and survival of robotic- versus video-assisted lobectomy in a prospective, randomized, blinded fashion in an international setting.

**Trial registration:**

ClinicalTrials.gov, NCT02617186. Registered 22-September-2015. https://clinicaltrials.gov/ct2/show/NCT02617186

## Introduction

### Background and rationale

Retrospective data demonstrates that robotic-assisted thoracoscopic surgery (RTS) provides highly precise instrumentation, 3-dimensional visualization, and a less steep learning curve as compared to video-assisted thoracoscopic surgery (VATS) [1], and RTS may also represent oncological benefit by allowing for improved lymph node dissection [2, 3]. Other potential advantages of RTS-Lobectomy over VATS-Lobectomy are decreased postoperative pain, lower mortality, shorter length of stay in hospital, shorter chest tube duration, and reductions in the incidence of common postoperative pulmonary complications [4–6]. Despite the potential benefits of robotic technology, there are two major barriers against its widespread adoption in thoracic surgery.

The first barrier is the lack of high-quality prospective data. To our knowledge, there are no prospective trials comparing VATS-Lobectomy to RTS-Lobectomy for early-stage lung cancer. In the largest multi-institutional series on RTS-Lobectomy with 325 patients, Park and colleagues reported a median length of stay in hospital of 5 days, 25% perioperative morbidity, 0.3% mortality, and 8% rate of conversion to thoracotomy [3]. Although these results compare favorably to most historical series on VATS-Lobectomy, this trial did not have a VATS-Lobectomy control arm. In a recent database analysis using State Independent Databases of 8 states, a propensity-matched cohort of RTS-Lobectomy was compared to VATS-Lobectomy [6]. Robotic resection was associated with reductions in mortality (0.2% vs 1.1%), length of stay in hospital (5.9 days vs 6.3 days), and overall complication rates (43.8% vs 45.3%) when compared with VATS.

The second major barrier to the widespread adoption of robotic technology in thoracic surgery is the perceived higher cost of RTS pulmonary lobectomy. In a recent trial of the Nationwide Inpatient Sample, it was determined that the incremental additional cost of RTS-Lobectomy over VATS-Lobectomy was $4,708 [7]. This dataset was limited by the absence of patient characteristics, the early learning curve for robotic surgeons, the large proportion of robotic cases being performed in community hospitals with small lobectomy volumes, and the lack of follow-up after discharge from hospital. Data on Health-Related Quality of Life (HRQOL) outcomes were most notably omitted from this trial, hampering any useful conclusion on the cost-effectiveness of RTS-Lobectomy.

In the face of these barriers, a randomized controlled trial comparing VATS-Lobectomy to RTS-Lobectomy is needed. Prospective randomization will eliminate the biases of retrospective data and will serve to determine whether there exists any advantages to HRQOL or patient outcomes in favour of RTS-Lobectomy over VATS-Lobectomy. Furthermore, through a prospective cost-utility analysis, this trial will provide the highest quality data to evaluate the true economic impact of robotic technology in thoracic surgery.

RTS-Lobectomy has only been recently introduced in Canada, and the volume of preliminary cases in our group (University of Toronto’s Toronto General Hospital (TGH) and McMaster University’s St. Joseph’s Healthcare Hamilton (SJHH)) is a reflection of this novelty. The first case was performed in May of 2011 at the TGH. In the first Canadian series of RTS for pulmonary resection for lung cancer, which included 167 completed cases performed at TGH and SJHH, analysis showed a median operative time of 270 minutes (233–326), a very low rate of conversion to thoracotomy (12/167, 7.2%) and a median hospital length of stay (LOS) of 4 days (3–6) [8]. To measure the effects of the learning curve, the data was stratified by surgeon and evaluated in temporal tertiles. Total operative time decreased significantly (p<0.001) over the learning curve; tertile 1: 309.0 min, tertile 2: 258.5 min and tertile 3: 236.0 min. Median time spent on the robotic console also decreased significantly (p<0.001) over tertiles– 172.0, 136.0, and 116.0 minutes, respectively [8]. Across tertiles, there were no differences in the median number of lymph node stations harvested (8, 8, 8; p = 0.39), length of stay (4, 4, 4; p = 0.16), or the rate of major intraoperative complications (Clavien-Dindo Class ≥ III; 8, 2, 8, respectively; p = 0.28). There were no mortalities.

The early Canadian experience with robotic lung cancer resection demonstrates excellent results that are comparable to those of experienced centers in operative times, length of stay and conversion rates, with further improvement demonstrated by the learning curve effect (Table 1).

**Table 1:** Outcome comparison between the first 167 cases of RTS-Lobectomy.

	Tertile 1	Tertile 2	Tertile 3
Operative time (minutes)	309.0	258.5	236.0
Time on robotic console (minutes)	172.0	136.0	116.0
Conversion (no., %)	2 (3.0)	2 (4.3)	7 (14.0)
Nodes harvested (no.)	8	8	8
Length of Stay (days)	4	4	4
Major Intraoperative Complications, Clavien-Dindo Class ≥ III (no.)	8	2	8
Mortality (no.)	0	0	0

VATS-Lobectomy is also a well-established procedure in our group. A recent analysis of our experience with 608 patients over the last 8 years demonstrated excellent rates of morbidity (26%) and 30-day mortality (0%), and a median length of stay in hospital of 4 days. More importantly, it was shown that the rate of lymph node sampling during VATS-Lobectomy performed by our surgeons is comparable to the rate of sampling during open lobectomy (Table 2). This is contrary to studies that question the oncological validity of VATS-Lobectomy because of poor rates of lymphadenectomy [9, 10], and demonstrates that our surgeons have reached an expert level of competency in this operation [3].

**Table 2:** Rates of lymphadenectomy in VATS-Lobectomy versus Thoracotomy in our group.

Nodal Station	Left Upper		Left Lower		Right Upper and Middle		Right Lower	
	VATS	Open	VATS	Open	VATS	Open	VATS	Open
2R					79%	77%	55%	52%
2L								
4R					95%	91%	73%	79%
4L	52%	64%	73%	74%				
5	64%	53%	50%	30%				
6	17%	6%	8%	11%				
7	73%	70%	97%	93%	94%	99%	90%	90%
8			17%	0%			7%	7%
9			79%	48%			21%	38%
10R					62%	46%	31%	38%
10L	47%	58%	54%	56%				
11R					72%	54%	69%	76%
11L	64%	70%	92%	81%				
12R					93%	79%	83%	93%
12L	87%	83%	54%	89%				

This trial will not include patients undergoing thoracotomy as a third comparator arm for two reasons. First, the superiority of RTS-Lobectomy and VATS-Lobectomy over thoracotomy has been consistently demonstrated in multiple high-quality studies [3–5], thereby eliminating clinical equipoise on this question. Second, in the present era of minimally invasive thoracic surgery, patients who undergo thoracotomy typically present with more advanced disease, and will thereby introduce a selection bias that will be difficult to mitigate.

### Objectives

#### Primary objective

Phase A of this trial is intended to determine the difference in patient-reported HRQOL outcomes between RTS-Lobectomy and VATS-Lobectomy at 12 weeks post-surgery. We hypothesize that for patients with early-stage non-small cell lung cancer (NSCLC), RTS-Lobectomy results in improved patient-reported quality of life as compared to VATS-Lobectomy.

#### Secondary objectives

In Phase A of this trial, we will compare differences in short-term clinical outcomes, and HRQOL outcomes at weeks 3 and 7; and months 6 and 12, which coincide with the intervals of oncological surveillance. We will also compute resource utilization and calculate the incremental cost effectiveness between RTS-Lobectomy and VATS-Lobectomy.

In Phase B of this trial, we will compare the difference in the 5-year survival data between the two arms. We will also compare differences in HRQOL outcomes at months 18 and 24; and years 3, 4, and 5, which coincide with the intervals of oncological surveillance. We will also compute resource utilization and calculate the incremental cost effectiveness between RTS-Lobectomy and VATS-Lobectomy.

RTS-Lobectomy is a new technology that has not been studied extensively yet against VATS-Lobectomy, so equipoise still exists. However, based on preliminary data from our experience and from literature, we hypothesize that for short-term clinical outcomes, RTS-Lobectomy leads to higher quality pathological staging by improved lymphadenectomy, and is associated with shorter duration of chest tube drainage; shorter hospital length of stay; less intraoperative blood loss; lower postoperative analgesia requirements; and less chronic postsurgical pain as compared to those who receive VATS-Lobectomy.

### Trial design

**Fig 1:**
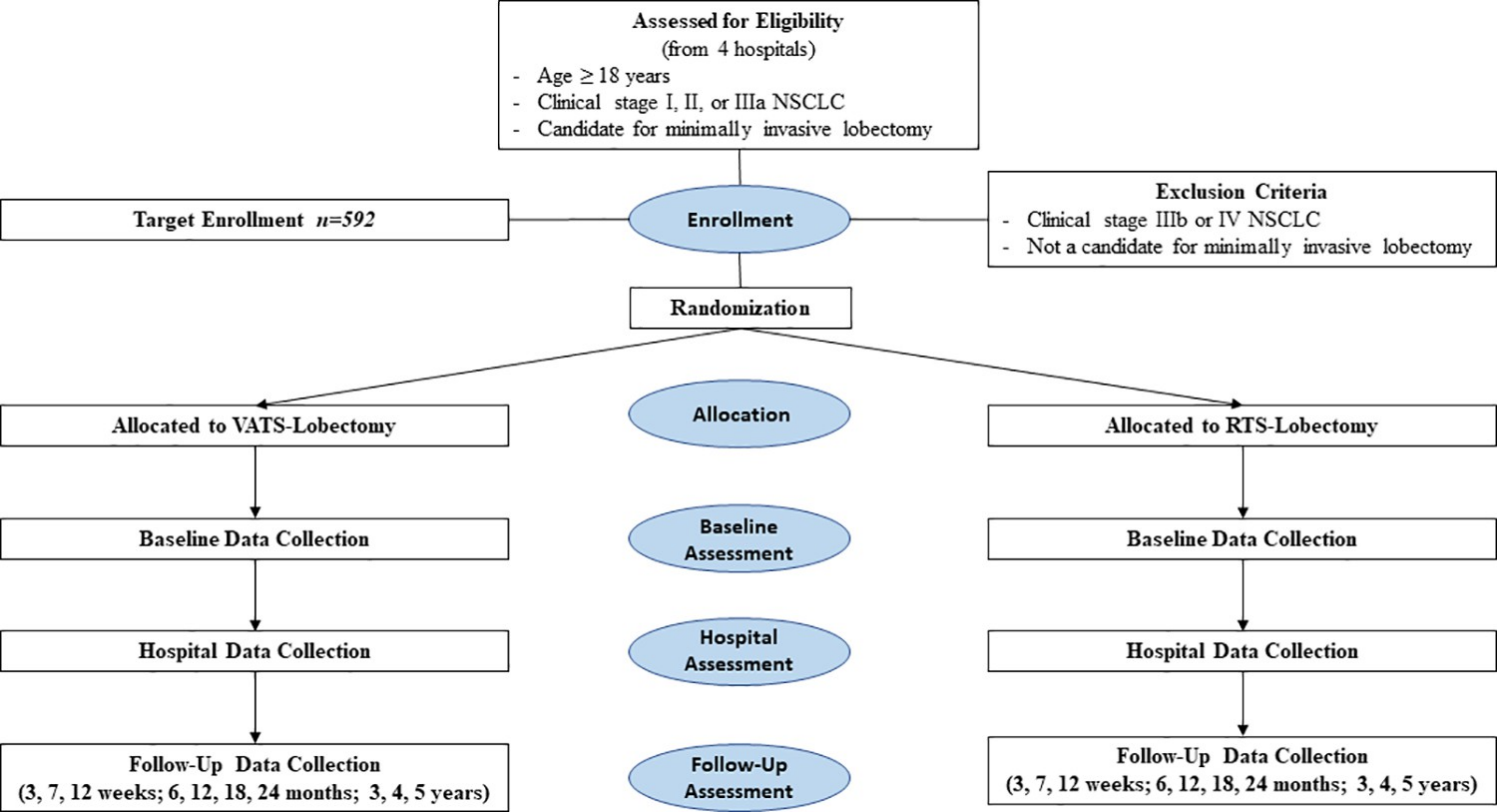
CONSORT diagram of trial design.

The trial is an international, multi-centered, blinded, randomized controlled trial. Patients will be randomized to RTS-Lobectomy versus VATS-Lobectomy at a 1:1 allocation ratio (Fig 1).

## Methods: Participants, interventions and outcomes

### Study setting

In Canada, participants will be recruited from two academic hospitals: McMaster University’s St. Joseph’s Healthcare Hamilton (SJHH) in Hamilton, Ontario and the University of Toronto’s Toronto General Hospital (TGH) in Toronto, Ontario. In Canada, these two hospitals have the highest volume for robotic thoracic surgery. Participants will also be recruited from two international academic hospitals: University of Florida’s UF Health Shands Hospital in Gainesville, Florida, USA, and Rouen Normandy University’s CHU-Hôpitaux de Rouen in Rouen, France.

### Eligibility criteria

#### Participants

Participants are eligible to participate in the trial if they sign the Patient Information and Informed Consent Form (PICF) and meet the following eligibility criteria:

*Inclusion criteria*:

- Age ≥ 18 years
- Clinical stage I, II or IIIa non-small cell lung cancer (NSCLC)
- Candidates for minimally invasive pulmonary lobectomy, as determined by the operating surgeon.

*Exclusion criteria*:

- Patients who received neoadjuvant treatment
- Clinical stage IIIb or IV NSCLC
- Not a candidate for minimally invasive lobectomy

During the COVID-19 pandemic, participants will be eligible to participate in the trial if they meet the eligibility criteria and provide either written or verbal consent.

#### Surgeons

The trial may be subject to performance bias, based on surgeon preference. To mitigate this, only surgeons who have demonstrated proficiency by independent completion of more than 30 VATS- and 30 RTS-Lobectomies [11] will be able to recruit participants for the trial.

### Who will take informed consent?

The investigator will introduce the trial at the time of consent for the operation. Under the guidance of the investigator, the trial coordinator will inform the potential trial participant of all pertinent aspects of the trial. Potential trial participants will be informed that their medical care will not be affected should they choose not to participate. The trial coordinator will encourage and answer any and all questions. Prior to participation in the trial, the trial participant, trial coordinator, and the investigator will print their name, sign, and date two copies of the PICF–one for the participant and one for the study team.

During the COVID-19 pandemic, the trial coordinator will inform the potential participant of all pertinent aspects of the trial by phone. Prior to participation in the trial, the PICF will be discussed over the phone. If the potential participant has an email address and access to a printer and scanner, then the PICF will be emailed to the potential participant for him or her to print, sign, scan, and email back. If the potential participant does not have an email address, nor access to a printer and scanner, then verbal consent will be recorded if provided. The consent discussion information will be recorded on the Consent Discussion Form.

### Additional consent provisions for collection and use of participant data and biological specimens

This is not applicable as the participant data collected will only be used for this trial. Biological specimens are not collected for this trial.

## Interventions

### Explanation for the choice of comparators

Both RTS-Lobectomy and VATS-Lobectomy are minimally invasive, standard of care surgeries. Despite the many potential benefits of RTS-Lobectomy, there are a couple of major barriers against its widespread adoption in thoracic surgery: lack of high-quality prospective data, and the perceived higher cost of RTS-Lobectomy. To the best of our knowledge, there is no prospective randomized controlled trial comparing these two types of minimally invasive surgeries, nor is there any useful conclusion on the cost-effectiveness of RTS-Lobectomy, and so we intend to compare RTS-Lobectomy and VATS-Lobectomy in this prospective, randomized controlled trial to overcome these barriers.

### Intervention description

The surgical procedure in both the intervention arms will involve pulmonary lobectomy and mediastinal lymph node sampling of stations 10R, 11R, 4R, 7, 8, 9 for right sided resections, and stations 10L, 11L, 4L, 5, 6, 7, 8, 9 for left sided resections. Each participant’s surgery will be videotaped.

In addition, participants in the:

*VATS-Lobectomy Arm*:

- will undergo the procedure through a 4-port technique with or without an accessory incision and,

*RTS-Lobectomy Arm*:

- will undergo the procedure according to the CPRL-4 technique (using 3 or 4 arms) as described by Cerfolio [12].

### Criteria for discontinuing or modifying allocated interventions

During the surgical procedure, if it is determined by the surgeon that in order to complete the surgery safely and successfully, the participant needs to be converted from their allocated intervention to a thoracotomy, then the allocated surgery will be discontinued. The participants will remain in their allocation group for purposes of intention-to-treat analysis.

### Strategies to improve adherence to interventions

The participants who enroll into this trial would be, as determined by the operating surgeon, candidates for minimally invasive pulmonary lobectomy. Hence, the participants should be able to undergo the surgery they were allocated to. However, above all else, the participants’ safety comes first, and if need be, then the surgeon will discontinue the allocated surgery for the participants’ safety.

Also, each participant’s surgery will be videotaped with a camera in the operating room. Any identifying features of the participant, including their face, will not be videotaped. There will be no audio on the videotapes. Videotapes will be reviewed by a member of the Video Review Committee for quality assurance purposes.

### Relevant concomitant care permitted or prohibited during the trial

As this trial is intended to determine the difference in outcomes between RTS-Lobectomy and VATS-Lobectomy, any concomitant care that is relevant and/or necessary during the participants’ standard of care during the trial will be permitted.

### Provisions for post-trial care

There are no trial-related provisions for ancillary or post-trial care. If a participant is injured or harmed directly from participating in the trial, all necessary medical treatments will be made available to them at no cost. Financial compensation for such things as lost wages, disability, or discomfort due to this type of injury or harm is not routinely available.

### Outcomes

#### Primary outcome

The primary outcome is difference in HRQOL scores between the treatment groups, as measured by the EQ-5D-5L questionnaire at week 12.

#### Secondary outcomes

To determine:

- Short-term clinical outcome differences, the following will be collected: clinical staging, pathological staging, number of lymph nodes sampled, admission date, date of surgery, discharge date, chest tube removal date, intraoperative blood loss, post-operative analgesia, and post-surgical pain.
- Resource utilization, a healthcare resource utilization tracking system will be developed for the specific purpose of the trial in order to allow for an accurate cost analysis. Variables that will be tracked include utilization of operating room time, operating room staff, surgical instruments and consumables, admission to critical care beds, hospital length of stay, duration of intravenous analgesia, postoperative complications, and costs associated with chronic post-surgical pain up to one year after surgery.
- Cost-Effectiveness, the incremental cost per quality-adjusted life year (QALY) gained will be calculated.
- Differences in HRQOL scores between the treatment groups, the EQ-5D-5L questionnaire will be administered at weeks 3 and 7; months 6, 12, 18, 24; and years 3, 4, and 5, which coincide with the intervals of oncological surveillance.
- Overall survival, the difference in 5-year survival rate between the two groups will be calculated.

### Participant timeline

**Fig 2:**
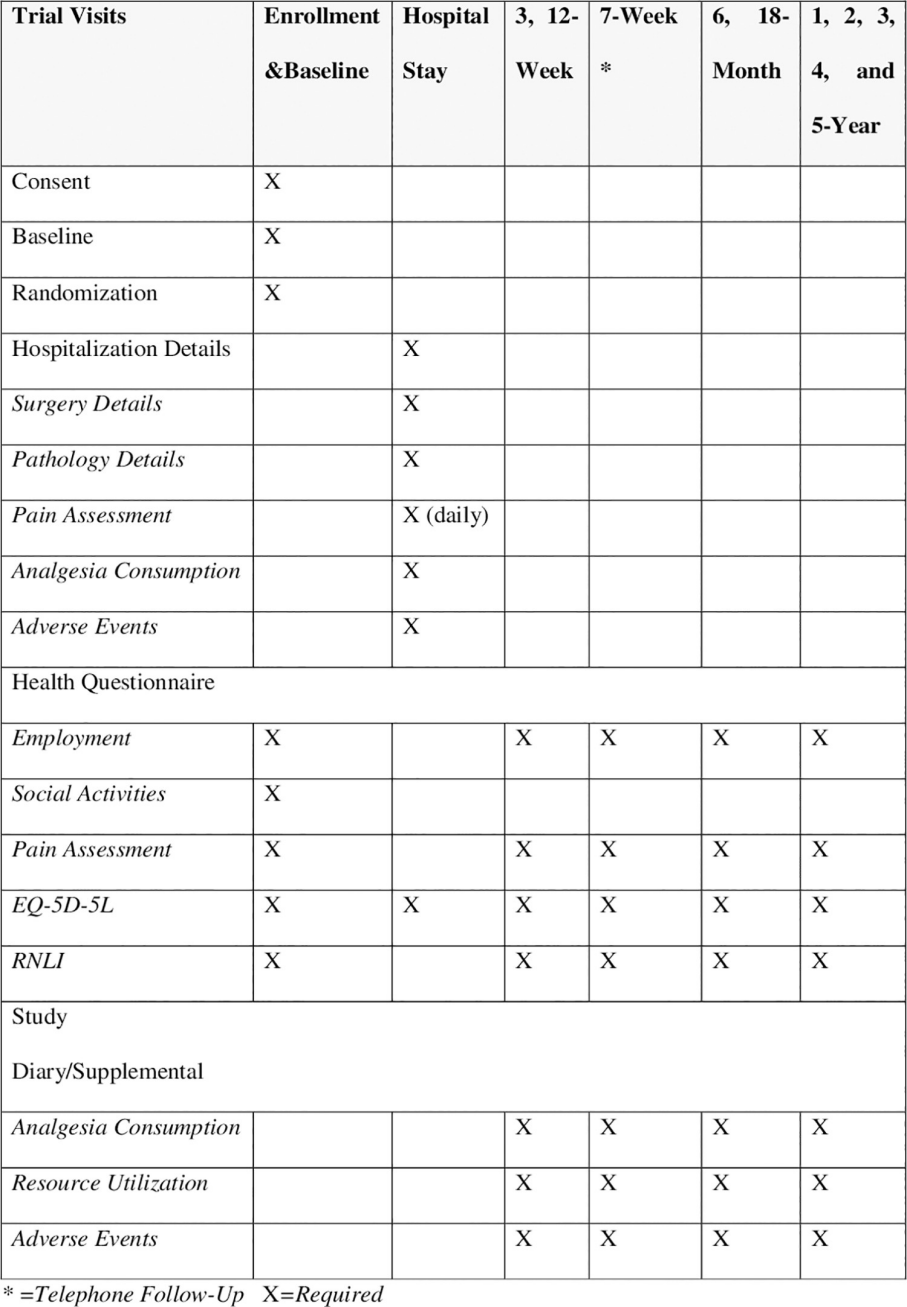
Schedule of assessments and evaluations.

The participant timeline of assessments is summarized below (Fig 2):

#### Enrollment and baseline

Potential participants will be screened to determine if they meet the eligibility criteria. Potential participants who meet the eligibility criteria will be approached by a trial coordinator to determine if they agree to participate. If they agree, they will be asked to and sign the PICF. Once the PICF is signed, they will be considered enrolled, and the participant will be randomized, asked to complete the Trial Health Questionnaire and be provided a Study Diary.

During the COVID-19 pandemic, the potential participants who have given permission to the PI to be contacted by a member of the research team, and who meet the eligibility criteria, will be approached via phone to determine if they agree to participate. If the potential participants have an email address, then the PICF will be emailed to them. The entire PICF will be reviewed and discussed. If they agree, they will be asked if they have a printer and scanner, so that the emailed PICF can be printed, signed, scanned, and emailed back to the research team. If they do have all of the listed items, then they will provide written consent. If they do not have all of the listed items, then they will provide verbal consent. Once either written or verbal consent is provided, they will be considered enrolled and then randomized. The consent discussion information will be recorded on the Consent Discussion Form.

#### Hospital stay

The participant will be admitted to the hospital and they will have the intervention they were randomized to. Intravenous patient-controlled analgesia will be administered to all patients in the hospital postoperative period, and managed by the hospital’s pain service. Prior to discharge, participants will complete the Trial Health Questionnaire.

#### Follow-up assessment

Follow-up will occur at weeks 3, 7, and 12; then months 6, 12, 18, and 24; and then years 3, 4, and 5 from the date of hospital discharge. All follow-up assessments, except for the 7-week, will take place in the hospital clinic at the time of the participant’s routine follow-up visits with the surgeon. The trial coordinator will be available to assist the participant in completion of the Trial Health Questionnaire as well as review the Study Diary with the participant. At the 7-week trial follow-up assessment, the trial coordinator will collect the data over the telephone. Five-year survival data will also be obtained by conducting a quick chart review at five-years. The patients have routine surveillance follow-up appointments with the surgeon every 6 or 12 months after surgery, either in-person or virtually, so survival data will be available from the surgeon’s surveillance follow-up note at five years.

### Sample size

#### Primary outcome in Phase A

The sample size for the primary outcome in Phase A was calculated based on the Minimally Important Difference (MID) of the EQ-5D-5L derived utility scores among lung cancer patients in the United States of America (USA). The index-based score is typically interpreted along a continuum where 1 represents full health and 0 represents dead, with some health states being worse than dead (<0). A MID of 0.07 and SD of 0.16 is considered clinically significant for this patient population [13]. The MID of 0.07 was considered clinically significant for this patient population because in 2007, Pickard et al. had reported that for EQ-5D utility scores in the USA, MIDs, grouped by performance status, ranged from 0.07 to 0.09 for all cancers and for lung cancer, but when based on FACT-G quintiles, MIDs were 0.06 to 0.07 in all cancers, and 0.05 to 0.06 in lung cancer [13]. A sample size of 166 patients, with 83 patients per arm will ensure detection of this difference with 80% power at a level of significance of 0.05. A 10% drop out rate was used to account for those potential withdrawals, therefore a total of 186 participants will be recruited to the trial, 93 participants per trial arm.

#### Secondary outcome in Phase B

Differences in QALY will require a higher number of measurements to be detected. Overall survival at 5-years will also require a larger sample size over a longer period of observation. These secondary outcomes of interest require the trial to be repowered so that they can be adequately powered. Based on a hypothesized 10% 5-year survival difference between the control arm and the experimental arm, the estimated sample sizes for two-sample comparison of survivor functions using the Log-rank test Freedman method with 80% power at a level of significance of 0.05 is 592 patients, with 296 patients per arm. A drop-out rate is not factored into this analysis since the Freedman method of censoring was employed, accounting for loss of patients over time. Also, an assumption for Stage I patients was made during this sample size calculation, since the majority of patients who are operated on are Stage I. Also, this calculation is not a non-inferiority design. This larger, sufficient sample size will allow comparison of difference in overall cost-effectiveness and survival.

### Recruitment

Historical institutional volumes at both Canadian centers demonstrate a total of 8–10 RTS-Lobectomies and 10–12 VATS-Lobectomies performed each month. A relatively high recruitment rate (~70–80%) is expected, due to public enthusiasm towards robotic surgery in Canada. The estimated completion of accrual is within 15 years of the trial start date.

## Assignment of interventions: Allocation

### Sequence generation

A statistician who is a member of the Biostatistics Unit at St. Joseph’s Healthcare Hamilton, generated the randomization sequence using SAS [14]. Randomization was stratified by surgeon. For each surgeon, a unique randomization sequence using the random permuted-block design (with blocks of varying sizes) to randomize patients in a 1:1 ratio to one of two intervention arms, VATS-Lobectomy or RTS-Lobectomy, was generated. This method ensures that an approximately equal number of patients will be allocated to each treatment group.

### Concealment mechanism

All participants who provide consent for participation and who fulfil all eligibility criteria will be randomized. Participants will be randomized using the central web-based Research Electronic Data Capture (REDCap) [15] randomization module. A statistician not otherwise involved with the trial will generate the randomization sequence and upload it into the REDCap randomization module. The module sequentially assigns participants to treatment groups and monitors the progress of allocation. The randomization sequence and blocking information will be concealed from all trial staff including the principal investigator, the biostatistician, and trial coordinators until the database is closed.

### Implementation

The trial coordinator will enroll participants in the trial. Once a participant is enrolled, the trial coordinator will access REDCap to randomize participants to either VATS-Lobectomy or RTS-Lobectomy. After a participant has been randomized, the allocation information will be stored in the database and will not be able to be modified.

## Assignment of interventions: Blinding

### Who will be blinded

As this is a surgical trial, provider blinding will not be feasible. Participants will remain blinded to the type of surgery they receive from the time of enrollment until the end of the trial follow-up period, at which time the trial coordinator will inform the participant as to which intervention they were randomized to (VATS-Lobectomy or RTS-Lobectomy). The operating room setup and resulting scars from both surgical procedures are comparable and should not compromise participant blinding. The biostatistician performing the analysis will be blinded as to which intervention arm participants were allocated to as the group allocations will be coded as Group A and Group B. To reduce observer bias, the trial coordinator (outcome assessor) will only provide minimal assistance (e.g., reading a question) to participants while they are completing their self-reported Trial Health Questionnaire.

### Procedure for unblinding if needed

At the end of the trial follow-up period, the trial coordinator will inform the participant as to which intervention they were randomized to (VATS-Lobectomy or RTS-Lobectomy).

## Data collection and management

### Plans for assessment and collection of outcomes

#### Data collection

This trial will include baseline, hospital, and follow-up (weeks 3, 7, 12; months 6, 12, 18, 24; and years 3, 4, and 5) assessments. Data for the trial will be gathered from the participants, electronic health records, and surgeon’s office chart as well as hospital databases (e.g., costing database). In addition, participants will be asked to complete the Trial Health Questionnaires and a Study Diary. Fig 2 provides a summary of the schedule of assessments and evaluations being conducted.

#### Baseline data collection

Baseline data including demographics (e.g., age, BMI, gender), medical history (e.g., smoking, alcohol use, comorbidities, analgesia consumption, pain, previous thoracic surgery, previous cancer), tumour characteristics (e.g., clinical stage, histology), and physiology (e.g., pulmonary function) will be collected. In addition, participants will be asked to complete the Trial Health Questionnaire and they will be provided with a Study Diary.

#### Hospital stay data collection

Hospitalization data including hospital admission date, hospital discharge date, chest tube removal date, intraoperative blood loss, and postsurgical pain will be collected. During the hospital stay, the pain will be assessed with the Numeric Pain Rating Scale (NPRS) on movement (deep breathing or mobilization) and analgesic consumption will be collected daily starting with postoperative day 1, until the date of discharge, and the videotape of the surgery will be collected. Pathology details will be collected including the number of lymph nodes harvested, pathology stage and histology. Direct costing data will also be collected (e.g., equipment used, surgical disposables used, staff in operating room, number of days in step down or intensive care unit, medications used). In addition, prior to discharge, participants will be asked to complete the Trial Health Questionnaire.

#### Follow-up assessment data collection

At the 3-, 7-, 12-week, 6-, 18-month, and 1-, 2-, 3-, 4-, and 5-year trial follow-up assessments, participants will be asked to complete the Trial Health Questionnaire and review the Study Diary with the trial coordinator. This is estimated to require 15 minutes of time. Five-year survival data will also be obtained by conducting a quick chart review at five-years. The patients have routine surveillance follow-up appointments with the surgeon every 6 or 12 months after surgery, either in-person or virtually, so survival data will be available from the surgeon’s surveillance follow-up note at five-years. The only survival data that will be collected at five-years is the status of the patient at five years (alive or not).

#### Trial health questionnaire

The Trial Health Questionnaire includes questions on the participant’s employment status, social activities (e.g., smoking, alcohol use), pain assessment (Numeric Pain Rating Scale) and two surveys, the EQ-5D-5L [16] and the Reintegration to Normal Living Index (RNLI) [17].

The NPRS is a numeric version of the visual analog scale, with 0 representing no pain and 10 representing the worst pain the participants can imagine. Participants will be asked to indicate their pain on movement (deep breathing or mobilization). The EQ-5D-5L has been validated for a number of chronic conditions, including cardiovascular and respiratory disease. The EQ-5D-5L is comprised of a descriptive system as well as a visual analog scale that assesses dimensions related to mobility, self-care, usual activities, pain/discomfort, and anxiety/depression. Respondents are asked to report their health states by rating each dimension as one of five levels (no problems, slight problems, moderate problems, severe problems and extreme problems). The RNLI is a self-report questionnaire used to evaluate global function status of a patient during rehabilitation which was developed to qualitatively assess the ability of individuals with traumatic or incapacitating illnesses to reintegrate into normal life. The RNLI is a questionnaire that is comprised of 11 questions that assesses mobility, self-care, daily activity, recreational activity, and family roles. Reintegration to normal living was defined as the reorganization of physical, psychological, and social characteristics of an individual into a harmonious whole so that one can resume well-adjusted living after incapacitating illness or trauma [18]. Each domain contains a visual analogue scale. On one end: “does not describe my situation” (1 or minimal integration) and “fully describes my situation” (10 or complete integration). Individual item scores are summed to provide the total score. The higher the score, the better the patients perceived integration.

#### Study diary

The Study Diary is designed to provide a space for participants to record any adverse events they may have, healthcare resources they may use (e.g., emergency room visits, hospitalizations, doctors and specialist visits, test, procedures or surgeries) and to record any pain medications they were prescribed and taking. At each visit the trial coordinator will review the Study Diary with the participant and ask any additional supplemental questions to clarify information recorded in the Study Diary.

### Plans to promote participant retention and complete follow-up

Participants will be informed that they are free to withdraw from the trial at any time, without fear of any negative repercussions. Investigators may also withdraw participants from the trial (e.g., to protect the participants’ safety). All trial withdrawals will be documented by date and reason for withdrawal. If the participant chooses to withdraw prior to randomization, their data will be excluded from the trial. If a participant chooses to withdraw after randomization, their data will be included, unless otherwise requested.

There is minimal anticipated loss to follow-up, as the majority of the interval data (3 weeks, 12 weeks, 6 months, 18 months, 1, 2, 3, 4 and 5 years) will be collected at the same time that the participant has their standard scheduled post-surgery follow-up visit in clinic. The availability of a trial coordinator will ensure that the Trial Health Questionnaire is completed at each of these follow-up clinic visits. If a participant misses a follow-up visit, it will be considered a deviation from the protocol, but the participant will remain in the trial and all attempts to contact the participant to conduct the visit over the telephone will be made, even if the visit occurs late. Major deviations from the protocol will be documented (e.g., the nature of the deviation and rationale for the deviation) and the REB will be notified as per requirements.

### Data management

At each trial site, data will be collected from source documents and entered into the electronic Case Report Forms (eCRFs) on REDCap. The eCRFs are the primary data collection instruments for the trial. REDCap is a secure web application developed to optimize data collection and management for research databases. REDCap will allow authorized data abstractors to enter trial data into eCRFs on a password-protected server hosted behind the SJHH firewall. Access to the trial database will be controlled by the database administrator (the trial coordinator at the Central Coordinating Centre) with overall access to the REDCap system granted by a SJHH Super-User. Each abstractor will be given individual credentials, allowing for entered content to be associated with the applicable abstractor, which will assist with auditing procedures in the future. REDCap allows for electronic prompts (e.g., range checks, valid values, missing data) on the eCRFs, which will minimize errors and omissions at the time of data entry, and a Data Resolution Workflow module where data queries can be added in response to standard data edit checks for discrepancies, consistency checks against data already stored in REDCap, and protocol deviations to ensure the integrity of the trial data.

Videotapes will be transferred from the Toronto, Florida, and France sites using the SFTP (SSH File Transfer Protocol), which ensures all data transmitted is encrypted in transit. The data will be received into a secure server in the Central Coordinating Centre’s DMZ (demilitarized zone). All videotapes will be stored on a secure server at the Research Institute of St. Joseph’s Healthcare Hamilton.

### Confidentiality

All records identifying the trial participant will be kept confidential and, to the extent permitted by the applicable laws and/or regulations, will not be made publicly available. Participant confidentiality will also be maintained in all analyses and presentations. At the time of enrolment, each participant will be assigned a unique trial participant identification (PID) number. Relevant personal health information (PHI) (e.g., first and last name, date of birth, telephone number, medical record number) will be recorded in a Trial Code List which will link the PHI to the PID. Information in the Trial Code List will be used to obtain and collect participant trial data from health records, determine age and to complete follow-up telephone assessments. The PID number will be recorded in the eCRF and no PHI from the Trial Code List will be entered in the eCRF. The Trial Code List and REDCap will be password protected and stored on the SJHH Research Institute’s secure server and will be protected in accordance with local data protection laws.

### Plans for collection, laboratory evaluation and storage of biological specimens for genetic or molecular analysis in this trial/future use

Not applicable as biological specimens will not be collected as part of this trial.

## Statistical methods

### Statistical methods for primary and secondary outcomes

Baseline characteristics of both treatment groups will be compared to ensure homogeneity in the patient population. Descriptive statistics will be reported by treatment group where categorical variables will be reported as counts (percentages) and continuous variables as mean (standard deviation) or as median (25^th^ percentile, 75th percentile). All analyses of primary and secondary outcomes will be carried out based on the intention-to-treat principle. Unadjusted comparisons of continuous outcome measures will be carried out using an independent t-test or the Wilcoxon rank-sum test if the assumptions of the t-test were violated. Unadjusted comparisons of categorical outcome measures will be computed using the chi-squared test, or the Fisher’s exact test if any of the expected values in the contingency table is less than 5, or McNemar’s test. For adjusted analyses, multivariable logistic regression analyses will be used for binary outcomes and multivariable linear regression for continuous outcomes. The results of comparisons between groups will be presented as mean differences for continuous outcomes and relative risks or odds ratios for binary outcomes, with corresponding 95% confidence intervals and associated p-values. ANOVA will be used for repeated measures. Survival will be compared using the Kaplan-Meier method and cox proportional hazard models. Survival curves will be compared using the Log-rank test. P-values will be reported to three decimal places with p-values less than 0.001 reported as p < 0.001. All analyses will be performed using SAS. Table 3 provides a summary of the variables, measures and methods of analysis.

**Table 3:** Variables, measures and methods of analysis.

Variable/Outcome	Hypothesis*	Outcome	Method of Analysis
**PRIMARY**			
Patient-reported Health-Related Quality of Life (HRQOL) outcomes	Improved	HRQOL scores, measured by the EQ-5D-5L at 12 weeks	Examine the distributions/ Wilcoxon rank sum test/regression
**SECONDARY**			
Short-Term Clinical Outcomes			
*Pathological Staging*	Improved	Pathological stage	McNemar’s test
*Lymph nodes*	More	Number of lymph nodes harvested	Independent t-test, Wilcoxon rank sum test/linear regression
*Duration of chest tube drainage*	Shorter	Number of days between surgery and chest tube removal	Kaplan-Meier method, Cox regression
*Hospital length of stay*	Shorter	Number of days between admission and discharge	Kaplan-Meier method, Cox regression
*Intra-operative blood loss*	Less	Volume of Intra-operative blood loss (mL)	Independent t-test/Wilcoxon rank sum test/ linear regression
*Post-operative analgesia requirements*	Less	In-hospital consumption (days, type, amount)	Independent t-test/Wilcoxon rank sum test/linear regression
*Chronic post-surgical pain*	Less	In-hospital pain (daily, scale)	Independent t-test/Wilcoxon rank sum test/ANOVA
Patient-reported Health-Related Quality of Life (HRQOL) outcomes	Improved	HRQOL scores, measured by the EQ-5D-5L at 3 and 7 weeks, and 6 and 18 months, and 1,2, 3, 4 and 5 years	Examine the distributions/ regression/ANOVA
Resource Utilization and Cost Effectiveness	More	Resource utilization tracking system, EQ-5D-5L, Return to Normal Living Index (RNLI), and employment status at 12 months	Incremental cost per quality-adjusted life year (QALY) gained will be calculated
Overall Survival	Improved	5-year survival difference	Kaplan-Meier method and cox proportional hazard models. Survival curves will be compared using the Log-rank test
**SENSITIVITY**			
Patient-reported Health-Related Quality of Life (HRQOL) outcome	Improved	HRQOL scores, measured by the EQ-5D-5L at 12 weeks	Independent t-test/ linear regression with multiple imputation for missing data

*Expected that RTS-Lobectomy will improve or be more, less, fewer or shorter than VATS-Lobectomy for each variable/outcome.

#### Primary analysis

The HRQOL of the patients will be described using both the EQ-5D-5L descriptive system as well as the corresponding utility scores calculated using the Canadian EQ-5D-5L value set [19]. The effect size using the EQ-5D-5L utility scores will be used to compare the difference in HRQOL between the two treatment arms.

### Secondary analysis

- Short-term clinical outcomes will involve aspects of perioperative care:
  ○ A higher quality of pathological staging is expected to occur with participants who have RTS-Lobectomy. Data on clinical and pathological staging will be compared.
  ○ More lymph nodes are expected to be sampled for participants who have RTS-Lobectomy. The number of lymph nodes sampled for each patient will define the outcome measure.
  ○ A shorter duration of chest tube drainage is expected to occur for participants who have RTS-Lobectomy. This will be measured by the number of days between the date of surgery and the date of chest tube removal.
  ○ A shorter hospital length of stay is expected to occur for participants who have RTS-Lobectomy. This will be measured by the number of days between the date of hospital admission and the date of discharge.
  ○ Less intraoperative blood loss (mL) is expected to occur for participants who have RTS-Lobectomy. This will be measured by the volume of blood loss in mL intraoperatively for each patient.
  ○ Less post-operative analgesia requirements are expected to occur with participants who have RTS-Lobectomy. This will be measured by collecting the amount of analgesia consumed by each participant (e.g., type of medication used (e.g., PCA, epidural, intravenous, oral), the number of days the medication was used, and the amount used) in hospital.
  ○ Less post-surgical pain is expected to occur in participants who have RTS-Lobectomy. This will be measured by collecting the pain score for each participant daily during their hospital admission.
- Resource Utilization and Cost Effectiveness
  ○ The cost analysis will only involve aspects of intraoperative and postoperative care because the preoperative evaluation of patients in both groups is identical. For robotic costs, calculating depreciation and maintenance costs for robotic equipment, based on the percent utilization of robotic resources by the thoracic surgery service will be done. Total costs will be calculated using the natural units of the relevant health care resource used by each participant and the unit costs for each resource item. QALY will be calculated by the area under the curve method, using health utility measured by EQ-5D-5L and the corresponding time duration. The incremental cost per QALY gained is calculated using the difference in the total cost divided by the difference in mean QALYs between RTS- and VATS-Lobectomy. Sampling uncertainty will be handled using the nonparametric bootstrapping approach. Cost effectiveness curves will be used to calculate the probability of RTS-Lobectomy being more cost effective than VATS-Lobectomy in treating this patient population at a wide range of maximum willingness to pay thresholds.
- HRQOL
  ○ The HRQOL will be analyzed as per the primary outcome at weeks 3 and 7, and months 6 and 18, and 1-, 2-, 3-, 4- and 5-years.
- Survival Analysis
  ○ Time to event analysis models will be used to compare overall survival at 3 years and 5 years between trial arms.

### Interim analyses

There are no planned interim analyses.

### Methods for additional analyses (e.g., subgroup analyses)

Subgroup analyses are planned to determine the impact of each of the following subgroups on the effectiveness of RTS-Lobectomy: body mass index (BMI), tumour size, and institution. Our hypothesis is that patient-reported health-related quality of life outcomes will be improved in the RTS-Lobectomy group than the VATS-Lobectomy group for patients who have a higher BMI, a larger tumour size, and had their minimally invasive lobectomy at a high-volume institution because data has shown that the rate of conversion to a thoracotomy is lower in RTS-Lobectomy versus a VATS-Lobectomy for these subgroups [20–23]. Regression using two-way interactions between the two study arms (RTS-Lobectomy versus VATS-Lobectomy) and each subgroup variable will be conducted to examine subgroup effects.

### Methods in analysis to handle protocol non-adherence and any statistical methods to handle missing data

Intention-to-treat implies all participants randomized are included in the analysis based on the treatment they were randomized to. We will impute missing data using multiple imputation [24] for the primary outcome only.

### Plans to give access to the full protocol, participant level-data and statistical code

There are no plans for granting public access to the full protocol, participant-level dataset, or statistical code.

## Oversight and monitoring

### Composition of the coordinating centre and trial steering committee

#### Central coordinating centre

The BFCRS-RP is the Central Coordinating Centre for this trial. In collaboration with the Steering Committee, the BFCRS-RP is responsible for the overall design, coordination and monitoring of the trial execution–particularly with regards to the methodological aspects to ensure adherence to the trial protocol and International Conference on Harmonization–Good Clinical Practice (ICH-GCP) at the clinical sites. The BFCRS-RP will monitor the trial centrally through all phases from initiation to data collection to trial close out. Administrative documents will be collected (e.g., REB approvals, approved PICF, delegation of authority form, investigator qualifications, agreements); protocol aspects will be monitored (e.g., enrollment criteria met); and data quality will be monitored (e.g., variables with excess missing data or data queries) to identify potential issues at sites and implement solutions quickly.

The BFCRS-RP has developed the trial protocol, consent form, data collection forms, and trial materials (e.g., operations manual), and developed and managed the trial database, data quality control, and other day-to-day coordination activities. The BRCRS-RP is responsible to prepare summary information and reports to keep the Steering Committee informed of trial progress, problems that require resolution and make decisions.

In addition, the BFCRS-RP is a clinical site and therefore is responsible for ethics submissions, recruitment, randomization, data collection, and conducting follow-ups for participants at the Charlton Campus of St. Joseph’s Healthcare Hamilton.

#### Sub-sites

Each site investigator is responsible for ensuring the trial at their site is conducted by appropriately trained individuals. On-site monitors will review the following documentation:

- Trial Essential Documents: Each site should maintain an Investigator Site File, including REB approvals and amendments, delegation of authority form, trial communications, etc.
- Trial Participant File: This file includes source documents which provide evidence for the existence of the trial participant and substantiate the integrity of the data collected. Fifty percent of files will be reviewed to ensure that participants signed the PICF, all data queries are resolved on the final eCRF and personal identifying information on source documents was removed prior to being filed. In addition, 50% of primary outcome data and 10% of secondary outcome data reported on the eCRFs will be checked to ensure that data that were transcribed from source documents are consistent with the source documents or explanations for discrepancies have been provided.

#### Steering committee

The Steering Committee, comprised of the principal investigator, trial coordinators, clinical site investigators, anesthesiologist, health economist and statistician are responsible for the scientific integrity of the trial including the overall design, reporting and publication of the trial to ensure that the execution and management of the trial are of the highest quality. The Steering Committee convenes on a regular basis (e.g., every 3 months) to review trial progress, and specifically to discuss rates of accrual, major protocol deviations, adverse events and resolve any trial issues.

#### Video review committee

The Video Review Committee is comprised of thoracic surgeons who are experts in robotic thoracic surgery and/or video-assisted thoracic surgery. Members of this committee periodically review surgery videotapes for quality assurance purposes.

#### Analysis committee

Biostatisticians will be responsible for the analysis, interpretation, tables, and reporting of results.

### Composition of the data monitoring committee, its role and reporting structure

A data monitoring committee is not needed for this trial because both interventions are standard of care and the potential risks of the intervention are well documented in both arms. In addition, the surgery is a one-time procedure.

### Adverse event reporting and harms

All adverse events (AEs), serious and non-serious, occurring during the trial will be collected according to the ICH-Good Clinical Practice Guidelines. The trial period during which adverse events must be reported is defined as the period after consent is obtained to the end of the follow-up period. AEs will be reviewed by the clinical investigators involved in the trial and assessed for their seriousness, severity and relationship to treatment. All serious adverse events (SAEs) will be reported to the investigators’ local REB and to the BRCRS-RP. AEs and SAEs will be summarized on a regular basis and reported to the Steering Committee. All SAEs that are still ongoing at the end of the trial period will be followed to report a final outcome.

### Frequency and plans for auditing trial conduct

The investigator will permit trial-related audits and inspections by their REB, the Trial Sponsor, and government regulatory bodies by providing direct access to all trial-related documents (e.g., source data/documentations, regulatory documents, data collection instruments, trial data). All audits and inspections will be carried out giving due consideration to data protection and trial participant confidentiality. All personal information made available for inspection will be handled in the strictest confidence and in accordance with local data protection laws.

### Plans for communicating important protocol amendments to relevant parties (e.g., trial participants, ethical committees)

If a protocol amendment is required due to a modification (e.g., eligibility criteria, outcomes, new information obtained), the BFCRS-RP will prepare a communication indicating the required amendment and the reason for the amendment. The communication will also include revised documents and a summary of the modifications. Sub-sites will submit the protocol amendment using the communications document provided by the BFCRS-RP, and once REB approval is received for the amendment, a copy will be forwarded to the BFCRS-RP and the amendment will be implemented.

### Dissemination plans

Decisions regarding the presentation and publication of the results of the trial will be made by the Steering Committee. It is the intent of the Steering Committee to publish the findings in the form of a peer reviewed manuscript. After publication, the trial will be presented at conferences and participants, if requested, will receive a summary report of the results of the trial.

## Discussion

Recently, a similar trial called the RVlob trial, which was a single-site, open labelled prospective RCT, reported on short-term results, but did not include survival [25]. Another similar trial, the ROMAN trial, which is pending publication, was not a blinded trial, and also did not study survival. If successfully completed, the RAVAL Trial will have studied survival and cost-effectiveness of robotic lobectomy in a prospective, randomized, blinded fashion in an international setting.

### Trial status

Protocol Version Number: V7

Protocol Version Date: July 13, 2020

Date Recruitment Began: January 21, 2016

Approximate Date when Recruitment will be Completed: December 31, 2031

To date, this trial is recruiting at 4 sites worldwide, and target recruitment and follow-ups are almost complete for Phase A. Recruitment is continuing for Phase B, and we are looking to open this trial at more sites as well. The major amendments over the years have been to open the trial at more sites worldwide.

### Ethics approval and consent to participate

Ethics approval has been obtained from the Hamilton Integrated Research Ethics Board (HiREB) on January 19, 2016, and the HiREB Project number is 0816. Each centre has also obtained ethics approval from their local Research Ethics Board (REB), and a copy has been forwarded to the Central Coordinating Centre.

Written, informed consent to participate will be obtained from all participants. During the COVID-19 pandemic, participants will be eligible to participate in the trial if they meet the eligibility criteria and provide either written or verbal consent. If the participants have an email address, printer, and scanner available to them at their own home, then we will ask that the participants provide written consent. If an email address, printer, and scanner are not available to the participants, then the participants will be eligible to participate if they provide verbal consent.

## Supporting information

S1 Checklist Completed SPIRIT checklist for the RAVAL trial protocol. (PDF)

S1 Protocol REB-approved protocol for the RAVAL trial. (PDF)

## Abbreviations

AE: adverse event
BFCRS-RP: Boris Family Centre for Robotic Surgery–Research Program
BMI: body mass index
COVID-19: Coronavirus Disease 2019
DMZ: Demilitarized Zone
eCRF: electronic case report form
HiREB: Hamilton Integrated Research Ethics Board
HRQOL: health-related quality of life
ICH-GCP: International Conference on Harmonization–Good Clinical Practice
LOS: Length of Stay
MID: Minimally Important Difference
NPRS: Numeric Pain Rating Scale
NSCLC: non-small cell lung cancer
PHI: Personal Health Information
PICF: Patient Information and Informed Consent Form
PID: Participant Identification
QALY: Quality-Adjusted Life Year
REB: Research Ethics Board
REDCap: research electronic data capture
RNLI: Reintegration to Normal Living Index
RTS: robotic-assisted thoracoscopic surgery
SAE: serious adverse event
SD: Standard Deviation
SFTP: SSH File Transfer Protocol
SJHH: St. Joseph’s Healthcare Hamilton
TGH: Toronto General Hospital
USA: United States of America
VATS: video-assisted thoracoscopic surgery
